# The structure of Polycomb-repressed chromatin in the bithorax complex

**DOI:** 10.1186/1756-8935-6-S1-O22

**Published:** 2013-03-18

**Authors:** Sarah K Bowman, Aimee M Deaton, Heber Domingues, Welcome Bender, Robert E Kingston

**Affiliations:** 1 Department of Molecular Biology, Massachusetts General Hospital, and Department of Genetics, Harvard Medical School, Boston, MA, USA; 2 Department of Biological Chemistry and Molecular Pharmacology, Harvard Medical School, Boston, MA, USA

## 

The 300 kb of the *Drosophila* bithorax complex (BX-C) is the original model for studying Hox gene repression by Polycomb group proteins. Decades of genetic experiments have led to the hypothesis that Polycomb organizes BX-C chromatin differently in each of the abdominal parasegments, and the structure of the chromatin is critical for maintaining cell identity. Studying the molecular organization of chromatin in individual parasegments has been technically difficult. We solved this problem by developing a sorted nuclei ChIP-seq pipeline. In this system, transgenic embryos produce tagged nuclei in single parasegments. Using FACS, we sorted these tagged nuclei and performed small-scale ChIP-seq. Initial results show that a histone mark catalyzed by Polycomb group proteins, trimethylated histone H3 at lysine 27 (H3K27me3), covers less of the BX-C as we move from PS5 to PS7. The boundaries of the H3K27 methylation correlate precisely with previously identified CTCF binding sites, a protein known to act as a chromatin barrier. Correspondingly, a mark that correlates with gene activation, H3K4me3, appears over the transcription start sites of BX-C genes that are not associated with Polycomb histone methyltransferase activity. Going forward, we will assay the localization of Polycomb group proteins and many other chromatin regulatory proteins to gain a complete picture of the chromatin environment that regulates Hox gene expression in the developing embryo. Still, the current results offer a compelling first look at BX-C chromatin *in vivo*, and provide molecular support to a long-standing genetic hypothesis.

